# A common modular design of nervous systems originating in soft-bodied invertebrates

**DOI:** 10.3389/fphys.2023.1263453

**Published:** 2023-10-03

**Authors:** Ekaterina D. Gribkova, Colin A. Lee, Jeffrey W. Brown, Jilai Cui, Yichen Liu, Tigran Norekian, Rhanor Gillette

**Affiliations:** ^1^ Coordinated Science Laboratory, University of Illinois at Urbana-Champaign, Urbana, IL, United States; ^2^ Neuroscience Program, University of Illinois at Urbana-Champaign, Urbana, IL, United States; ^3^ Stanson Toshok Center for Brain Function and Repair, Rosalind Franklin University of Medicine and Science, North Chicago, IL, United States; ^4^ Department of Molecular & Integrative Physiology, University of Illinois at Urbana-Champaign, Urbana, IL, United States; ^5^ Whitney Laboratory for Marine Biosciences, University of Florida, St. Augustine, FL, United States

**Keywords:** evolution, homology, analogy, pallium, reticular system, *Pleurobranchaea*

## Abstract

Nervous systems of vertebrates and invertebrates show a common modular theme in the flow of information for cost-benefit decisions. Sensory inputs are incentivized by integrating stimulus qualities with motivation and memory to affect appetitive state, a system of homeostatic drives, and labelled for directionality. Appetitive state determines action responses from a repertory of possibles and transmits the decision to a premotor system that frames the selected action in motor arousal and appropriate postural and locomotion commands. These commands are then sent to the primary motor pattern generators controlling the motorneurons, with feedback at each stage. In the vertebrates, these stages are mediated by forebrain pallial derivatives for incentive and directionality (olfactory bulb, cerebral cortex, pallial amygdala, *etc.*) interacting with hypothalamus (homeostasis, motivation, and reward) for action selection in the forebrain basal ganglia, the mid/hindbrain reticular formation as a premotor translator for posture, locomotion, and arousal state, and the spinal cord and cranial nuclei as primary motor pattern generators. Gastropods, like the predatory sea slug *Pleurobranchaea californica*, show a similar organization but with differences that suggest how complex brains evolved from an ancestral soft-bodied bilaterian along with segmentation, jointed skeletons, and complex exteroceptors. Their premotor feeding network combines functions of hypothalamus and basal ganglia for homeostasis, motivation, presumed reward, and action selection for stimulus approach or avoidance. In *Pleurobranchaea*, the premotor analogy to the vertebrate reticular formation is the bilateral “A-cluster” of cerebral ganglion neurons that controls posture, locomotion, and serotonergic motor arousal. The A-cluster transmits motor commands to the pedal ganglia analogs of the spinal cord, for primary patterned motor output. Apparent pallial precursors are not immediately evident in *Pleurobranchaea*’s central nervous system, but a notable candidate is a subepithelial nerve net in the peripheral head region that integrates chemotactile stimuli for incentive and directionality. Evolutionary centralization of its computational functions may have led to the olfaction-derived pallial forebrain in the ancestor’s vertebrate descendants and their analogs in arthropods and annelids.

## Introduction

Did the general neural organization of decision and action selection precede or follow the evolution of complex body form and behavior? We argue that despite broad differences in complexity (detail) of brain and body, soft-bodied invertebrates like gastropods even with primitive, incompletely centralized brains show a blueprint for a modular organization of the nervous system similar to vertebrates, although simpler in detail. Vertebrates, insects, and polychaete annelids likely evolved segmentation and articulated skeletons with jointed appendages independently (Mullins et al., 2011; Hochner, 2013; Katz, 2016). These acquisitions are adaptations for speed and agility beyond what is easily gotten by a soft body. The skeletal joints allow local sensory-motor regulation of segmental musculature, and sensory monitoring of joint angles for incorporating into central motor templates. Vertebrates and insects share analogous modular designs for computational functions that mediate decision, spatial mapping, and motivation (Loesel and Heuer, 2010; Tosches and Arendt, 2013; Holland, 2016). Such functions may have originated in the ancestral bilaterian with simpler anatomy and behavior or could have evolved independently multiple times. The above lineages each have more complicated body forms than the expected urbilaterian ancestor (Hejnol and Martindale, 2008).

For comparative analysis, it is useful to examine circuits in organisms more like the expected common ancestor. It is pertinent that a simple sea slug, with primitive ciliary locomotion and lacking segmentation and appendages, has modular design in neuronal network coordination similar to vertebrates for posture, locomotion, and general arousal. Thus, the general scheme controlling locomotion and posture could have preceded the evolution of segmented bodies and articulated appendages. Could then the neuronal circuitry of cost-benefit decision and action selection be also analogous, and possibly homologous in origin, among the phyla?

**FIGURE 1:**
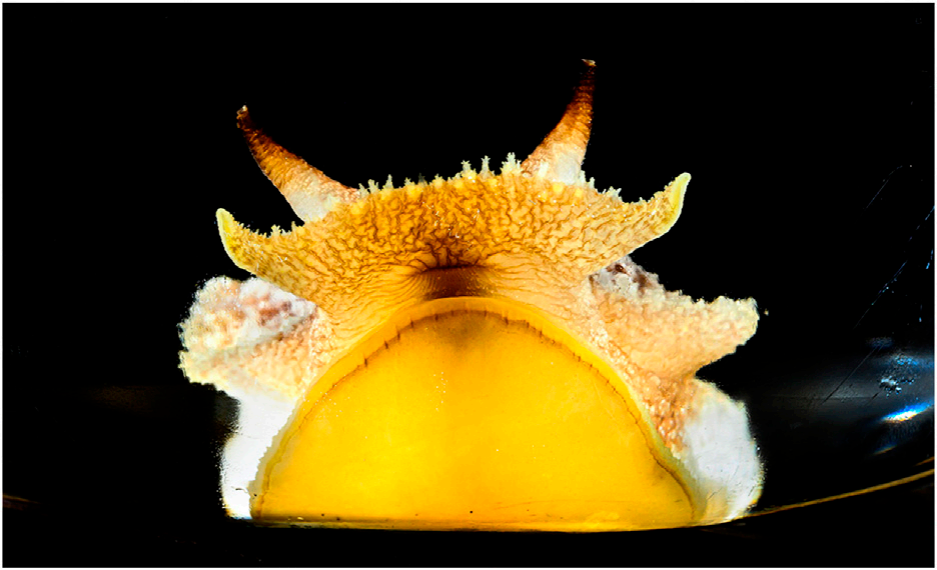
*Pleurobranchaea californica*, head shot. The chemotactile oral veil is flared above the mouth. The oral veil papillae and the lateral tentacles have ciliated chemotactile receptors that feed into a peripheral subepithelial network to mediate incentive and stimulus location. Photo by Fred Zwicky.

We previously discussed the evolution of the nervous system and developed hypotheses on the nature of the nervous system in the common urbilaterian ancestor (Gillette and Brown, 2015). As the ancestor is unavailable, we took a classic comparative approach. We used the sea slug *Pleurobranchaea californica* as a model system whose nervous system and behavior have been examined and are likely relatively close to the ancestor’s (Figure 1). The nervous system and behavior are also known in more detail than for many other animal systems, particularly with respect to mechanisms for decision-making, and are amenable to the comparative approach. Moreover, there is much useful information on brain and behavior of related gastropods. Among other model systems available, information on the brains and behaviors of vertebrate animals has been amassed over more than a century by thousands of researchers. Thus, we may compare markedly distant lineages that split over 500 Myr ago into protostome and deuterostome by examining functional analogies in brain and behavior.

Comparisons of functional analogies in brain and behavior of the streamlined *Pleurobranchaea* with the rather more complex vertebrates may show commonalities interpretable in terms of direct or parallel evolution. Gastropods like *Pleurobranchaea* boast systems that may be closer in brain, body, and behavior to the simple common ancestor than the more complex, segmented vertebrates, insects, and polychaete annelids with jointed appendages. The former lack complicating segmentation and jointed skeletons with appendages and many of them locomote primitively by myriads of cilia paddling their way through secreted mucus, as expected of the common ancestor. Moreover, especially for the opisthobranch sea slugs like *Pleurobranchaea*, their soft bodies and behaviors are quite simple, and they are hermaphroditic with minimal investment in offspring care, thereby further reducing neural complexities of animals with two different sexes that may brood their young.

*Pleurobranchaea* shows simple reward-based learning for approach or avoidance of prey (Noboa and Gillette, 2013). Their behavior and neuronal circuitry are characterized well enough to generate computational simulations which express cost-benefit decisions in autonomous agents with efficient, reward-based mechanisms for stimulus valuations. The algorithms used reproduce the basic modular functions of nervous systems as treated here (Brown et al., 2018; Gribkova et al., 2020).

Thus, a fuller answer to the nature and origin of complexity in human brain and behavior can begin to be sought in the simplicity of our very early ancestors. We may safely assume that the problems faced by the ancestors were basically the same as they are today, and that their nervous systems were designed by natural selection to generate appropriate behavioral algorithms. The threefold basic imperatives of life are: 1) acquisition of resources; 2) defense; and 3) reproduction. These three constraints of behavioral design are largely interconnected and interdependent. Even single cells may follow this design, where biochemical pathways do the computations of an integrating nervous system, connecting sensors to motor pathways for movement and secretion. We annotate these constraints briefly, below, and then continue on to document the organization of simple and complex nervous systems in resolving the imperatives.

### Acquisition of resources

The essential resource is energy, which underlies the abilities for defense and reproduction. More complicated faces of this basic resource come from derived characters aimed at maximizing and conserving energy, like shelter, clothing, and storage in fatty tissues. Even social characters such as attention to status and reputation, play, and aggression intimately involve the economics of energy. Other resources are the chemical building blocks of cells: amino acids, fats, carbohydrates, and water.

### Defense

Once life evolved to a point where it could acquire energy and use it in reproduction, strong selective forces would have had it evolve mechanisms to keep from losing it, such as a cell membrane (which then involved a whole new set of problems resulting in the evolution of ion pumps). Once organisms had developed to conserve energy, they became treasure troves to some that devised ways to break into their stores and use them for themselves. This would have been an early manifestation of predation.

### Reproduction

As early cells devised asexual and sexual ways of reproduction, impelled by the obvious selective forces excluding nonreproductive cells, reproductive predators evolved to hijack their mechanisms, becoming intracellular parasites, and perhaps degenerating from free-living cells to viruses (Forterre, 2006). Thus, the three design constraints are inextricably intertwined.

For most animal species that have been observed in detail, the cost-benefit decisions employed to solve the problems of acquisition, defense, and reproduction integrate internal state, sensory input, and memory. This holds for vertebrates, insects, and gastropod mollusks (see Gillette et al., 2000; Clore et al., 2021). The dynamic changes in internal state for motivation, in varying sensory input, and in memory plasticity may lead to a broad spectrum of cost-benefit responses tailored to each situation. There are sufficient data presently to compare the neural architectures that support such decision-making. First, we would like to review the functional modules of the vertebrate nervous system, then compare them with analogs in the sea slug.

In each of these functions there is a modular design for the flow of information from stimulus to response: incentive-directionality; reward expectation; approach-avoidance decision; decision implementation in motor response.

## Vertebrate nervous systems

A modular organization of the mammalian nervous system, in terms of interacting subsystems, has been long recognized both anatomically, physiologically, and computationally (Arbib, 2007).

### Hypothalamus

Nutrition, thermoregulation, hydration, and social behaviors like sex and parental care are homeostatic drives founded in the hypothalamus, whose neural activity represents appetitive state and biases behavioral choices for the different goals. Nutritional state is fairly well understood. In vertebrates, nutritional state is closely monitored by hypothalamic neurons sensitive to serum glucose, digestive hormones, and hormonal indicators of fat and glycogen storage. Motivation, in terms of hunger/satiation, regulates exploration after food resources or avoidance of such activity. Moreover, the hypothalamic circuits and hormones that mediate hunger and satiety appropriately regulate the sensitivity of the reward system (Cassidy and Tong, 2017).

### Basal ganglia

Action selection for cost-benefit decisions is determined by the basal ganglia, which integrate sensory incentives weighed with motivation-related reward and memory of experience (Turner and Desmurget, 2010). The basal ganglia have access to cortical areas with memories laden with positive and negative affect related to context and can compare possible costs and benefits of potential decisions. Final summation leads to selection of motor actions that essentially represent approach or avoidance of a predicted situation. The decision outputs are sent to premotor areas in the reticular system of the brainstem that extends from the upper part of the midbrain to the lower part of the medulla. Important dopaminergic and serotonergic modulatory inputs to the basal ganglia come from the substantia nigra compacta and raphe nuclei, respectively, for reward and arousal.

### Reticular formation and spinal cord

Premotor patterning networks in the hindbrain reticular system are disinhibited by basal ganglia inputs to form premotor commands configured for the selected motor actions. From the reticular formation the commands for locomotion and posture descend to the spinal cord to be translated into direct motor action by the segmental pattern generators. The reticular system has critical roles in maintaining arousal state in behavioral and motor systems through ascending pathways to the forebrain and descending pathways to the spinal cord via the reticulospinal tracts. The raphe nuclei of the reticular system are critical serotonergic sources for modulation of mood, reward, and arousal (Teissier et al., 2015; Venkatraman et al., 2017). Ascending fibers from the anterior raphe nuclei innervate the basal ganglia and other forebrain derivatives, and descending fibers from the posterior nuclei innervate the spinal cord pattern generators and sensory pathways.

The spinal cord is segmented, and the coupled spinal pattern generators’ final outputs are through their motorneurons. Spinal pattern generators receive direct feedback from muscle and tendon stretch receptors and from descending inputs that can smooth coordination and aid adaptation of motor pattern to uneven terrain (Rossignol et al., 2006).

### Pallial derivatives

Olfactory bulb, cortex, pallial amygdala, and other pallial structures are thought to have evolved as elaborations of the olfactory system, with the addition of more sensory inputs from complex exteroceptors such as mediate vision, audition, proprioception, and taste (Reiner et al., 1998; Redgrave et al., 1999; Smeets et al., 2000; Aboitiz et al., 2003; Grillner et al., 2013; Aboitiz and Montiel, 2015; Jacobs, 2023). Functionally, these structures interact with each other and the subpallial striatum to integrate reward, motivation, affect, and memory to compute incentive for decision making and to place it in physical context with directional information. The motor cortex apparently only appeared in evolution with mammals and is not treated further here.

### Analogs in the Gastropod’s nervous system

**FIGURE 2:**
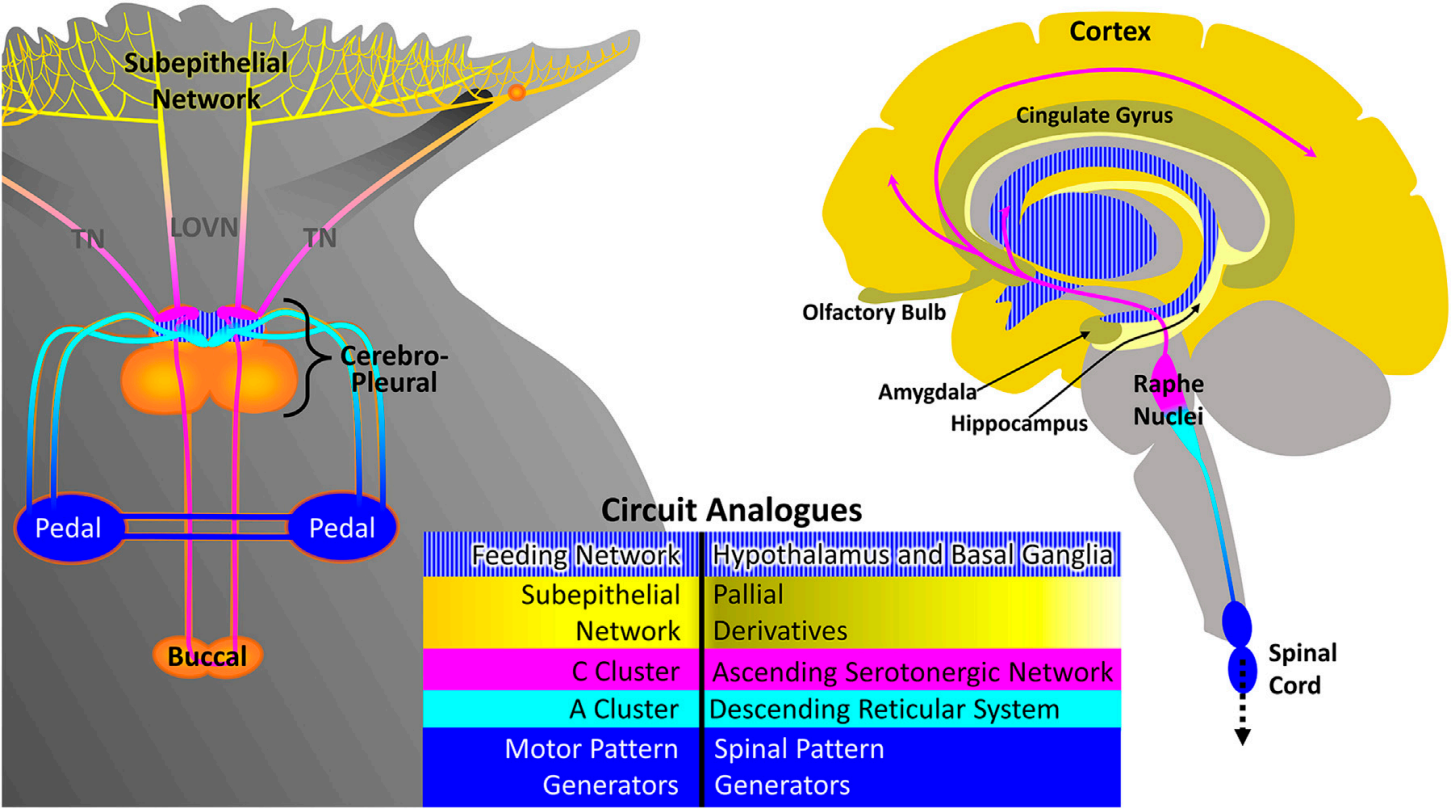
Proposed analogs of *Pleurobranchaea californica* and vertebrate central nervous systems, as represented by the human brain and spinal cord. The feeding network (blue striped) in the cerebral lobes of the cerebropleural ganglion combines homeostatic functions of the vertebrate hypothalamus in motivation and decisive action selection of the basal ganglia. The C-cluster (magenta) is functionally analogous to the serotonergic portions of the vertebrate ascending reticular system. The A cluster (cyan) is functionally analogous to the vertebrate reticular system, projecting to pattern generators in the pedal ganglion, which are in turn analogous to the mammalian spinal cord in final motor pattern output. The serotonergic cells of the A-cluster are analogous to the serotonergic cells of the descending raphe nuclei. The chemotactile sensory subepithelial network (yellow) of *Pleurobranchaea* is proposed as resembling an ancestral precursor to pallial derivatives of the ancient olfactory system in evolution, such as the olfactory bulb, neocortex, hippocampus, basal ganglia, amygdaloid complex and others.

Comparison of the gastropod decision-making system, represented by *Pleurobranchaea*, with vertebrates’ finds general similarities in organization (Figure 2) However, a major exception regarding vertebrate forebrain is discussed below. Mainly, they differ in complexity of detail related to the elaborate bodies and exteroceptors of the vertebrates. Previous work described neuronal circuitry underlying cost-benefit and approach-avoidance decisions in *Pleurobranchaea*’s foraging (Jing and Gillette, 2000; Gillette and Jing, 2001; Yafremava et al., 2007; Hirayama et al., 2012; Hirayama and Gillette, 2012; Brown, 2014). These circuits share functional analogies with the vertebrate striatum and hypothalamus in that they express homeostatic state and instruct goal-directed action selection (Gillette and Brown, 2015), with the analogies extending to coordination of locomotion in goal-directed behavior. Thus, a cluster of neurons, the A-cluster, works analogously to the reticular system for posture, locomotion, and arousal (Lee et al., 2023), and a peripheral neuronal network may serve functions of forebrain pallial derivatives for incentive and simple place-coding. Of the several ganglia of Pleurobranchaea’s central nervous system (CNS), the cerebropleural ganglion contains the circuits in its cerebral lobes that function analogously to the combined basal ganglia-hypothalamus and the reticular system. Sensory input to the ganglion is integrated with motivation and memory to decide actions. These decisions descend to pattern generators in the pedal ganglia, which themselves have functions analogous to the vertebrate spinal cord in final patterning of direct motor output (Figure 2). A significant difference may be that *Pleurobranchaea*’s basal ganglia analog has only the direct neural pathway output integrating sensation, internal state, and memory, and lacks the explicit comparison of potential costs and benefits afforded by the indirect pathway originating in the vertebrate striatum, as may the insects (Strausfeld and Hirth, 2013).

### Cerebral lobes: feeding network

The distributed feeding motor network combines the homeostatic functions of the hypothalamus in motivation and the basal ganglia in decisive action selection. Hunger/satiety is represented in the excitation state and neuronal activity configuration of the feeding network (Hirayama et al., 2012; Hirayama and Gillette, 2012; Hirayama et al., 2014). Sensory input incentivized by memory summates with the neuronal representation of motivation to bring the network towards or away from exploring a stimulus. In cost-benefit decisions for approach-avoidance, hungry animals approach food odors, but when satiated they turn away from them. The feeding network excitation state sets the thresholds for active feeding, which can vary a million-fold between ravenous hunger and logy satiation (Davis and Gillette, 1978; London and Gillette, 1986; Hirayama et al., 2014). Incentivized input to the feeding network may be mediated by dopaminergic afferents from chemotactile areas (Kabotyanski et al., 2000; Brown et al., 2018), which may well include the effects of learned preference and avoidance (Elliott and Susswein, 2002). The functions of *Pleurobranchaea*’s homeostatic feeding network in action selection are located in the subpallial basal ganglia of the vertebrate, however still under the influence of the hypothalamus analog of the feeding network. Thus, in evolution the computations of homeostasis and action selection may have been either separated to different modules in evolution in the vertebrates or joined in the feeding network of gastropods.

**FIGURE 3:**
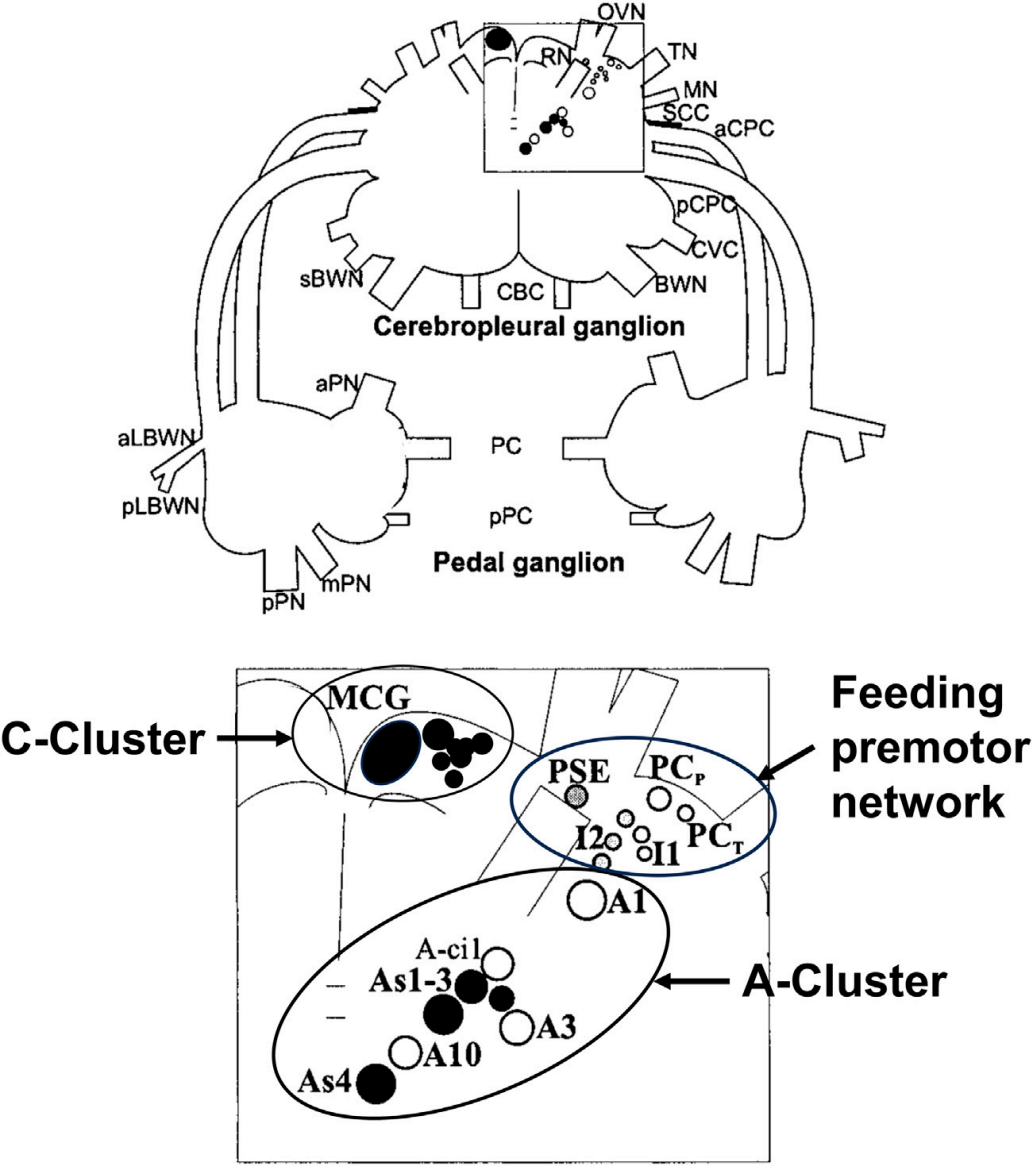
Drawing of the dorsal surface of the cerebropleural and pedal ganglia showing identified neuronal somata of networks for the feeding network, the A-Cluster for posture, locomotion, and motor arousal, and the serotonergic C-Cluster. Filled circles show serotonin immunoreactive somata. The somata are bilaterally symmetrical, but only shown unilaterally. Abbreviations: MCG, metacerebral giant neuron; PCP, phasic paracerebral interneuron; PSE, polysynaptic excitor of the PCP; PCT, tonic paracerebral interneuron; I1, I2, Interneurons 1; and 2, Interneuron 2; BWN, body wall nerve; sBWN, small body wall nerve; CBC, cerebrobuccal connective; aCPC, anterior cerebropedal connective; pCPC, posterior cerebropedal connective; CVC, cerebrovisceral connective; MN, mouth nerve; OVN, oral veil nerve; RN, rhinophore nerve; SCC, subcerebral commissure; TN, tentacle nerve; aLBWN, anterior lateral body wall nerve; pLBWN, posterior lateral body wall nerve; PC, pedal commissure; pPC, parapedal commissure; aPN, anterior pedal nerve; mPN, medial pedal nerve; and pPN, posterior pedal nerve.

Figure 3 shows a map of the neurons of the premotor feeding network, the A-cluster that controls posture and locomotion with its serotonergic As1-4 interneurons, and the anterior serotonergic C-cluster to which we will refer below.

**FIGURE 4:**
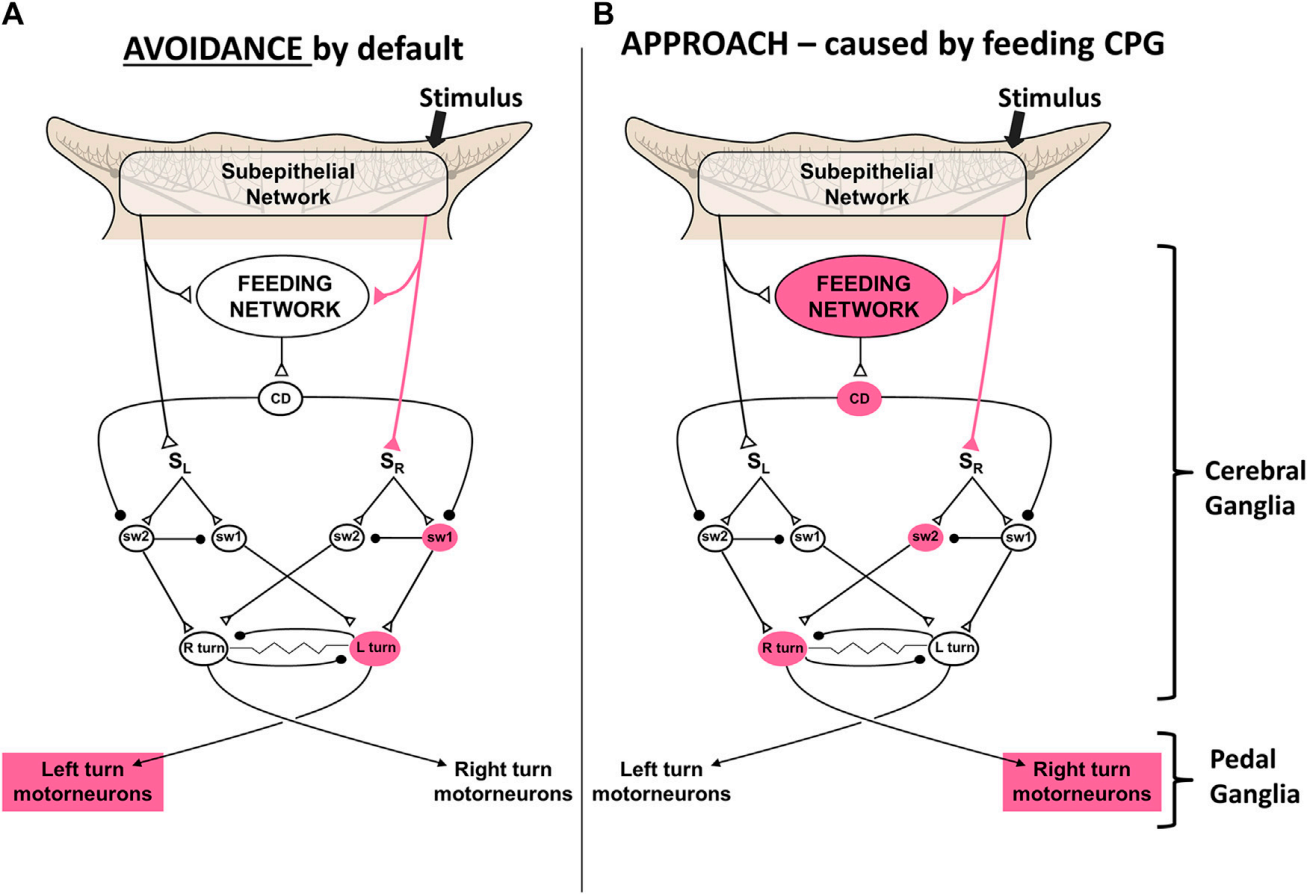
A simplest model for control of the turn by excitation state of the feeding motor network. A simple neuronal switch mechanism under control of corollary discharge from the CPG mediating feeding is postulated. **(A)** When the activity of the feeding network is low, sensory inputs from the oral veil drive avoidance turns away from the site of the stimulus. **(B)** Increased activity of the network, through hunger or greater sensory stimulation, changes the avoidance response to an approach-turn toward the stimulus by a postulated disinhibitory mechanism that shifts activity to the contralateral side of the turn motor network. Figure modified from Gillette and Brown (2015).

Action selections by the feeding network are quite simple choices between approach and avoidance of stimuli, and whether to locomote or not. Avoidance turns are the default responses to sensory stimuli in the absence of feeding network excitation. Higher activity in the feeding network converts the turn responses to approach, possibly by shunting sensory input to the turn network from one side to the other (Gillette et al., 2000; Hirayama and Gillette, 2012; Brown, 2014; Hirayama et al., 2014). Thus, partly satiated animals actively avoid weaker feeding stimuli and approach stronger ones. Active feeding causes inhibition of the premotor neurons that mediate turning and locomotion, which prevents the animal from overrunning its prey before finishing the meal. Figure 4 shows a simplest model based on synaptic disinhibition routing excitation to the opposite side of the turn motor network, reflecting similar downstream disinhibitory mechanisms by the basal ganglia in action selection.

### Cerebral lobes: reticular system analog

#### A-cluster

In *Pleurobranchaea*, the analog of the vertebrate reticular formation lies in a bilaterally paired group of neurons, the A-cluster (Jing and Gillette, 1999; Jing and Gillette, 2000; Lee et al., 2023), posterior to the feeding network neurons in the cerebral lobes. A-cluster homologs are also found in other gastropods (Sudlow et al., 1998; Popescu and Frost, 2002; Jing et al., 2008; Jing et al., 2009). The A-cluster was first recognized in a search for the premotor pattern generator for the escape swim. This search was correctly based on a presumption that the swim network would incorporate the neurons of the turn network, which could be studied for approach-avoidance decisions. Thus, it was eventually found that the A-cluster in different states of coordination mediates the escape swim, approach-avoidance turns, crawling locomotion, and likely righting behavior (Jing and Gillette, 1995; Jing and Gillette, 1999; Jing and Gillette, 2000; Jing and Gillette, 2003). Thus, A-cluster neurons are concerned with different aspects of posture and locomotion, very similar to the functions of the reticular system. The major differences in complexity can be simply related to control of a soft body vs. an articulated, skeletonized body.

Moreover, as for the raphe nuclei of the reticular formation, the behavioral functions of the A cluster are critically dependent on a serotonergic network. The four bilaterally paired serotonergic As1-4 neurons lie at the core of the A-cluster and provide the neuromodulatory drive to swimming, turning, and crawling locomotion. They are interconnected both chemically by serotonergic synapses and electrically, and fire in different patterns with the different motor outputs. Unlike the concentrated localization of the ∼7 raphe nuclei in the vertebrate brain, serotonergic neurons of the gastropod brain are dispersed within and among the ganglia, lying within motor pattern generators like the A-cluster and the As1-4 cells and the feeding network and the C-cluster, where they drive motor arousal (Jing and Gillette, 2003; Gillette, 2006). However, their interconnections allow activity in one group to contribute to the arousal of another and thereby its motor network. If the serotonergic neurons of the gastropod were indeed homologous to those of the vertebrate raphe nuclei, it could mean either that the serotonergic neurons became concentrated in vertebrates or that they dispersed in the mollusks (Gillette, 2006).

Like the posterior raphe nuclei, the serotonergic As1-4 neurons send descending innervation to the pedal ganglia analogs of the spinal pattern generators to drive crawling locomotion and approach-avoidance turns in different states of coordination (Jing and Gillette, 2003; Lee et al., 2023). They are also chemically coupled to the serotonergic neurons of the feeding motor network. In coordination with other identified neurons of the A cluster, they sustain the swimming escape response episode (Jing and Gillette, 1999). Thus, their actions are obligatory to the actions of posture and locomotion mediated by the A-cluster analog of the reticular activating system.

#### C-cluster

The serotonergic C-cluster neurons and the metacerebral giant neurons of the cerebral ganglia lobes innervate the feeding motor network and the peripheral sensory neuronal network. They are functionally analogous to the serotonergic portions of the vertebrate ascending reticular formation (Figure 2), which innervates the hypothalamus, basal ganglia, and other pallial derivatives. The C-clusters of 12+ neurons each and the metacerebral giant neurons lie anteriorly in the cerebral lobes and are both electrically and chemically coupled (Gillette and Davis, 1977; Jing and Gillette, 2000). They are embedded in the feeding network where during the active feeding cycle they receive alternating synaptic excitation and inhibition during the retraction and protraction (swallowing and biting) phases of the feeding apparatus, respectively (Gillette and Davis, 1977). The C-cluster cells richly innervate the cerebral ganglia lobes and send axons to the periphery to innervate the subepithelial network (see below) of the chemotactile oral veil (Moroz et al., 1997), where ongoing work indicates that serotonin promotes sensory gain. The paired metacerebral neurons provide neuromodulatory input to the buccal ganglion’s feeding central pattern generator (CPG), but the majority of its outputs are peripheral: to the esophagus/crop, the musculature of the buccal mass, and the chemosensory mouth area (Gillette, 1991). Serotonergic modulation of the feeding network sets the basal excitation state and lowers the thresholds for both the approach turn and active feeding (Hirayama and Gillette, 2012; Hirayama et al., 2014). Serotonergic modulation of sensory gain in the gastropod peripheral nervous system may contribute to incentivization of stimuli and conceivably also contributes to attentional mechanisms and odor/taste learning.

#### Pedal ganglia–spinal cord analogs

Like the vertebrate spinal cord, the gastropod pedal ganglia mediate the larger part of direct motor patterning for posture and locomotion. They receive descending commands, including neuromodulatory serotonergic input, from the A-cluster in *Pleurobranchaea*, to express the motor outputs of approach and avoidance turns, escape swimming, body withdrawal, and locomotion. They correspond to the subesophageal ganglia of other ecdysozoans like the arthropods and annelids, which partially serially segment in development to form the ventral nerve cords, themselves similar to vertebrate spinal cord in development and molecular signatures (Vergara et al., 2017).

#### Subepithelial network–potentially like the ancestral pallial precursor?

The pallial circuitry of the vertebrate brain is largely concerned with processing incentive on the basis of stimulus qualities, motivation, reward experience (hedonic affect), and memory, and with directional information for stimuli. *Pleurobranchaea* has no such central neuronal module that has yet been identified. However, there is a peripheral, subepithelial network (SeN) of interneurons postsynaptic to the primary receptors for chemotactile stimuli in the oral veil that does perform simple functions of incentivizing stimuli and estimating their directional location (Figure 2). Thus, it is quite possible that the origin of the telencephalic derivatives of the ancient olfactory system in evolution–the olfactory bulb, cortex, hippocampus, basal ganglia, amygdaloid complex and the others–may have lain in a peripheral SeN processing incentive and directionality like that in the oral veil of *Pleurobranchaea*, which became centralized and enhanced with the evolving body complexities of segmentation and jointed skeletons.

Olfaction is among the most primitive of senses (Cuschieri, 1976). Elaboration of olfactory systems and associated structures for odor learning is closely tied to the emergence of pallial structures. Directional olfaction may have formed the basis for the evolution of memory organization in the bilaterian brain, with hippocampal-like structures used for mapping and encoding the spatial distributions of novel odorants (Jacobs, 2012; 2023). Further, the olfactory system is the only sensory system that bypasses the thalamus, a structure for multi-modal sensory relay; instead, the olfactory bulb projects directly to amygdala and piriform cortex, which are important for emotion and memory (Kay and Sherman, 2007; Jacobs, 2023). Like the olfactory bulb, the SeN of *Pleurobranchaea* is notably dopaminergic and is richly innervated by the serotonergic neurons of the C-cluster.

The primary olfactory centers in vertebrate and insect brains seem to have similar neuronal types, sensory coding mechanisms, coherent oscillatory activity, and organization, including dense olfactory projections to structures critical for memory: vertebrate hippocampus and insect mushroom bodies (Strausfeld and Hildebrand, 1999; Tomer et al., 2010). Some gastropods share similar features, including presence of glomeruli and coherent oscillations in procerebral lobes of pulmonate mollusks, which are important for odor learning (Gelperin, 1999; Kay, 2015).

In *Pleurobranchaea*, while there is no clear analogue to mushroom bodies or procerebral lobes, learning and mapping of olfactory stimuli are supported by the oral veil SeN. Computations in the SeN allow *Pleurobranchaea* to map locations of environmental stimuli relative to its body. The cell bodies whose axons carry the location information have central cell bodies (unpublished observations). This somatotopic map of stimulus location and distribution over a target area can provide a template for directed motor reaction, and further, it offers a potential evolutionary substrate type for place coding, contextual memory and spatial memory, as seen in arthropod mushroom and ellipsoid bodies, and vertebrate hippocampus (Devaud et al., 2015; Eichenbaum, 2017).

The SeN suggests insights into the evolution of learning and memory circuits by its nature as an extensive interconnected sensory network with dopaminergic elements that also receives prominent central serotonergic innervation. The interactions of dopamine and serotonin are notable in other systems for supporting hetero-associative reward feedback underlying attention and short- and long-term memory. A basic form of associative learning is classical conditioning, where a neutral stimulus is paired with a biologically potent stimulus, such as food, reward, or even pain, to elicit a conditioned response. The simplest of associative memory systems that can support classical conditioning must have circuitry that handles at least two different kinds of input, such as a sensory signal and motor feedback signal, or a sensory signal paired with a reward feedback signal (e.g., Hawkins et al., 1998). Second-order conditioning involves the associative pairing of a neutral stimulus with an already conditioned (previously neutral) stimulus, to elicit a conditioned response (Brogden, 1939). Circuitry for second-order conditioning must therefore contain a neuron or set of neurons that responds to both types of neutral stimuli.

Thus, the higher in complexity a memory system is, the more intermediate circuitry it must have between neurons that respond to sensory input and those that receive reward or motor feedback. This is particularly apparent in systems capable of sequence learning and spatial memory, such as mammalian hippocampus and octopus’ vertical lobe, which have both extensive auto-associative and hetero-associative architectures. The SeN expresses potential precursors to these associative architectures as it consists of an extensive interconnected sensory network that also receives intricate serotonergic innervation, which could well provide hetero-associative reward feedback.

Spatial learning demands neuronal circuitry that can associate the order and strength of sequentially encountered stimuli, with their connectivity influenced by reward. In principle, this does not look complicated. Such learning may have been selected for in evolution in motile opportunists for efficiently exploiting particular territories, and for homing to a safe refuge when not foraging. While the nomadic and homeless *Pleurobranchaea* most likely lack spatial learning, they provide an interesting example of a potential neuronal precursor for more context-dependent substrates of associative learning. In particular, the *Pleurobranchaea* SeN is well-positioned for associatively pairing specific odor and reward stimuli, and it may even support pairing of the *location* of the specific olfactory stimulus relative to its body with reward feedback. This possible encoding of additional context, i.e., the somatotopic stimulus location, may enable the simple short-term working odor memory that may aid *Pleurobranchaea* in navigating its olfactory environment (Yafremava and Gillette, 2011). A recurrent inhibitory network was proposed as a simple averaging computation of stimulus loci for motor targeting (Yafremava and Gillette, 2011). Moreover, the serotonergic innervation of the SeN by the C-cluster cells could well provide a mechanism for working memory, attention, and long-term memory through heterosynaptic facilitation.

## Conclusion

**FIGURE 5:**
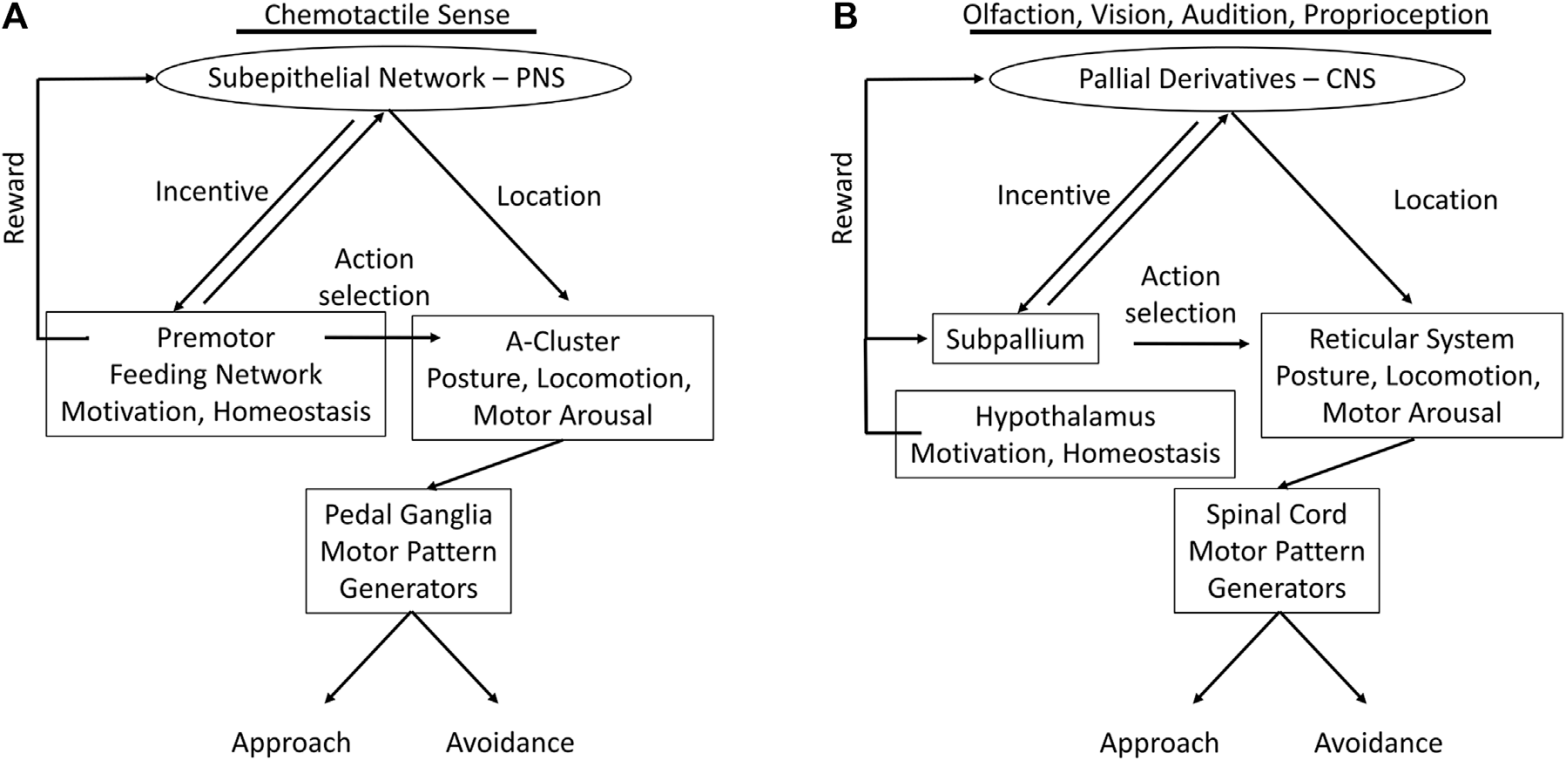
Functional analogs in nervous systems of *Pleurobranchaea* **(A)** and vertebrates **(B)**; comparing the flow of information from sensory input to motor output. In the vertebrates the incentive and directionality functions of the gastropod subepithelial network are centralized and markedly enhanced in pallial cortex (entorhinal, hippocampal, *etc.*) for vision, audition, and posture. Action selection for approach-avoidance decisions in the vertebrates, which in *Pleurobranchaea* is the province of the feeding network, is separated to the subpallial basal ganglia. The motivational and homeostatic functions of the *Pleurobranchaea* feeding network are retained by the vertebrate hypothalamus. The multifunctional premotor network in the A-cluster neurons of *Pleurobranchaea* is analogous to the vertebrate reticular formation, framing action decisions in terms of motor arousal, posture, and locomotion. The pedal ganglia and spinal cord receive motor commands from the premotor networks and express the primary motor patterns.

We have summarized marked analogies between the complex vertebrate systems, and to some degree those of insects, with the nervous systems of gastropods much simpler in body form and behavior. Figure 5 recapitulates the functional analogies, possible homologies and/or convergences, between the nervous systems of the gastropod *Pleurobranchaea* and vertebrates.

The evolved neural complexities of the vertebrates and insects may be largely ascribed to their segmentation and jointed skeletons, which lend them speed and agility through ability to monitor body posture for motor planning, to more functional equilibrium organs, and to their high resolution exteroceptors for vision, vibration, and electroreception in some cases. However, we are able to describe a similar modular organization of the nervous system in the gastropod *Pleurobranchaea*, though so much simpler in body form and behavior, with many fewer neurons, and even incomplete centralization of the CNS. Figure 5 summarizes the analogous neural modules between *Pleurobranchaea* and vertebrates that mediate action selection and reward as feeding network/basal ganglia and hypothalamus. The analogies for posture, locomotion, and motor arousal are the neuron group A-cluster/reticular system of the mid-to-hindbrain. For the serotonergic raphe nuclei of the vertebrate reticular system, the *Pleurobranchaea* analogy is a distributed serotonergic network with ascending and descending projections, and for primary motor pattern generation, the spinal cord is analogous to the *Pleurobranchaea* pedal ganglia.

Finally, we introduced the SeN as a simplest analog of the pallial derivatives (olfactory bulb, cortex, *etc.*). The SeN receives interconnected input from primary sensory receptors to compute incentive from stimulus qualities and to estimate the averaged stimulus source direction (place) for directed motor response. Its final output targets are at the feeding network, where it sums with appetitive state, and at the turn motor network in the A cluster, where it sets the moment-to-moment amplitude of the approach-avoidance turn. The computations of incentive and stimulus location lend the SeN qualities which could have been precursor to the more detailed computations of the vertebrate forebrain for incentive, for the computations of more complicated place codes of more environmental stimuli transduced by complex eyes and ears, and for the eventual evolution of more complex cognitive ability in episodic memory for signatures in space and time.

In the vertebrates, these stages are mediated by pallial derivatives for incentive (olfactory bulb, cerebral cortex, amygdala, &c) interacting with hypothalamus (motivation and reward) for action selection. The reticular formation acts as a premotor translator for posture, locomotion, and arousal state, and the spinal cord and cranial nuclei as primary motor pattern generators.

Finally, our work here may contribute to an answer to whether the modular organizations of vertebrate and arthropod brains evolved independently or were inherited and embellished from a soft-bodied, unsegmented bilaterian ancestor. The marked similarities observed in gastropods are consistent with a common inheritance and may invite future comparative molecular and developmental analyses to cast further light on this compelling question.

## Data Availability

The original contributions presented in the study are included in the article/Supplementary Material, further inquiries can be directed to the corresponding author.
